# Osteoclasts modulate the balance between immunosuppression and inflammation through antigen presentation and interactions with CD4^+^ T cells

**DOI:** 10.1186/1479-5876-9-S2-P56

**Published:** 2011-11-23

**Authors:** Grazia Abou Ezzi, Thomas Ciucci, Vanessa Amiot, Abdelilah Wakkach, Claudine Blin-Wakkach

**Affiliations:** 1 U576 INSERM, Hopital l’Archet, Nice, France

## Background

Osteoclasts (OCLs) are bone resorbing cells derived from the monocyte lineage (MN-OCLs) in steady state special. We have shown that in vivo, dendritic cells can differentiate into OCLs (DC-OCLs) and this mechanism requires the presence of inflammatory CD4^+^ T cells. Recent studies have suggested that MN-OCLs could modulate T cells. Our aim was to better characterize the effect of OCLs on T cells and in particular to compare both OCL subsets (DC-OCLs and MN-OCLs) in terms of antigen presentation and T cell activation.

## Material and methods

DC-OCLs and MN-OCLs were generated in vitro from murine bone marrow. After purification, their phenotype and effect on T cells were analyzed in coculture in an antigen-specific system.

## Results

Our results revealed that both OCL subsets express similarly the molecules involved in antigen presentation and costimulation. Both OCL subsets process and present antigens identically, without the need to resorb. Transcriptomic analyses showed that MN-OCLs express higher levels of immunosuppressive cytokines whereas DC-OCLs express higher levels of inflammatory cytokines. Their effect on T cells was evaluated in coculture of DC-OCLs or MN-OCLs with CD4+ T cells from OTII mice (ovalbumin-specific TCR) in the presence or not of ovalbumine. Using CFSE staining, we found that DC-OCLs induce T cell proliferation more efficiently than MN-OCLs. Intracytoplasmic analysis of cytokine production revealed that DC-OCLs induce efficiently a Th1 polarization whereas MN-OCLs induce regulatory T cells.

## Conclusion

Our results demonstrate that OCLs may have an immunomodulatory function. cells. The immunosuppressive effect of MN-OCLs may maintain a tolerance against self-antigens produced during bone resorption. The immunogenic capacity of DC-OCLs may participate to the vicious circle linking bone destruction to chronic inflammation. This mechanism represents a very novel concept in the regulation of the balance between immunosuppression and inflammation.

